# Pretreatment Vitamin D Concentrations Do Not Predict Therapeutic Outcome to Anti-TNF Therapies in Biologic-Naïve Patients With Active Luminal Crohn’s Disease

**DOI:** 10.1093/crocol/otad026

**Published:** 2023-05-15

**Authors:** Neil Chanchlani, Simeng Lin, Rebecca Smith, Christopher Roberts, Rachel Nice, Timothy J McDonald, Benjamin Hamilton, Maria Bishara, Claire Bewshea, Nicholas A Kennedy, James R Goodhand, Tariq Ahmad

**Affiliations:** Gastroenterology, Royal Devon University Healthcare NHS Foundation Trust, Exeter, UK; Exeter Inflammatory Bowel Disease and Pharmacogenetics Research Group, University of Exeter, Exeter, UK; Gastroenterology, Royal Devon University Healthcare NHS Foundation Trust, Exeter, UK; Exeter Inflammatory Bowel Disease and Pharmacogenetics Research Group, University of Exeter, Exeter, UK; Gastroenterology, Royal Devon University Healthcare NHS Foundation Trust, Exeter, UK; Exeter Inflammatory Bowel Disease and Pharmacogenetics Research Group, University of Exeter, Exeter, UK; Gastroenterology, Royal Devon University Healthcare NHS Foundation Trust, Exeter, UK; Exeter Inflammatory Bowel Disease and Pharmacogenetics Research Group, University of Exeter, Exeter, UK; Biochemistry, Exeter Clinical Laboratory International, Royal Devon University Healthcare NHS Foundation Trust, Exeter, UK; Biochemistry, Exeter Clinical Laboratory International, Royal Devon University Healthcare NHS Foundation Trust, Exeter, UK; Gastroenterology, Royal Devon University Healthcare NHS Foundation Trust, Exeter, UK; Exeter Inflammatory Bowel Disease and Pharmacogenetics Research Group, University of Exeter, Exeter, UK; Gastroenterology, Royal Devon University Healthcare NHS Foundation Trust, Exeter, UK; Exeter Inflammatory Bowel Disease and Pharmacogenetics Research Group, University of Exeter, Exeter, UK; Exeter Inflammatory Bowel Disease and Pharmacogenetics Research Group, University of Exeter, Exeter, UK; Gastroenterology, Royal Devon University Healthcare NHS Foundation Trust, Exeter, UK; Exeter Inflammatory Bowel Disease and Pharmacogenetics Research Group, University of Exeter, Exeter, UK; Gastroenterology, Royal Devon University Healthcare NHS Foundation Trust, Exeter, UK; Exeter Inflammatory Bowel Disease and Pharmacogenetics Research Group, University of Exeter, Exeter, UK; Gastroenterology, Royal Devon University Healthcare NHS Foundation Trust, Exeter, UK; Exeter Inflammatory Bowel Disease and Pharmacogenetics Research Group, University of Exeter, Exeter, UK

**Keywords:** vitamin D, IBD, Crohn’s disease, PANTS

## Abstract

**Background and Aims:**

Vitamin D has a regulatory role in innate and adaptive immune processes. Previous studies have reported that low pretreatment vitamin D concentrations are associated with primary non-response (PNR) and non-remission to anti-TNF therapy. This study aimed to assess whether pretreatment 25-hydroxyvitamin D concentrations predicted PNR and non-remission to infliximab and adalimumab in patients with active luminal Crohn’s disease.

**Methods:**

25-Hydroxyvitamin D concentrations were measured in stored baseline samples from 659 infliximab- and 448 adalimumab-treated patients in the Personalised Anti-TNF Therapy in Crohn’s disease (PANTS) study. Cut-offs for vitamin D were deficiency <25 nmol/L, insufficiency 25–50 nmol/L, and adequacy/sufficiency >50 nmol/L.

**Results:**

About 17.1% (189/1107; 95% CI, 15.0–19.4) and 47.7% (528/1107; 95% CI, 44.8–50.6) of patients had vitamin D deficiency and insufficiency, respectively. 22.2% (246/1107) of patients were receiving vitamin D supplementation. Multivariable analysis confirmed that sampling during non-summer months, South Asian ethnicity, lower serum albumin concentrations, and non-treatment with vitamin D supplementation were independently associated with lower vitamin D concentrations. Pretreatment vitamin D status did not predict response or remission to anti-TNF therapy at week 14 (infliximab *P*_pnr_ = .89, adalimumab *P*_pnr_ = .18) or non-remission at week 54 (infliximab *P* = .13, adalimumab *P* = .58). Vitamin D deficiency was, however, associated with a longer time to immunogenicity in patients treated with infliximab, but not adalimumab.

**Conclusions:**

Vitamin D deficiency is common in patients with active Crohn’s disease. Unlike previous studies, pretreatment vitamin D concentration did not predict PNR to anti-TNF treatment at week 14 or nonremission at week 54.

## Background

By binding to the vitamin D receptor expressed on most immune cells, vitamin D has a key regulatory role in innate and adaptive immune processes. Relevant to the pathogenesis of inflammatory bowel disease (IBD), in animal and *in vitro* experimental models, vitamin D modulates tight junctions, maintaining intestinal epithelial integrity and regulating host–microbiota interactions.^1^

Patients with IBD have multiple risk factors for vitamin D deficiency including chronic diarrhea, bile salt malabsorption, dietary restrictions, and reduced sunlight exposure. Consequently, vitamin D deficiency is more common than in the general population,^2–4^ and while it does not cause IBD,^5–8^ because of the link with active disease,^9–12^ there is considerable interest in the role of vitamin D as an adjunct to IBD therapies.^13^

Over the last 3 decades, the anti-TNF monoclonal antibodies, infliximab and adalimumab, have become the most frequently prescribed biologics for immune-mediated inflammatory diseases. Unfortunately, anti-TNF treatment failure in patients with Crohn’s disease is common: One-quarter of patients experience primary nonresponse, one-third of responders lose response, and only 40% of patients are in remission at the end of a year.^14,15^ Anti-TNF monotherapy, obesity, smoking, disease severity, and the development of antidrug antibodies are associated with low drug concentrations and subsequent anti-TNF treatment failure.^15,16^ Carriage of the HLA-DQA1*05 allele confers a 2-fold risk of developing antibodies to anti-TNF treatment.^17,18^

In small retrospective studies, vitamin D deficiency has been associated with primary non-response, non-remission, and durability of anti-TNF therapy.^19–21^ We sought to assess whether pretreatment 25-hydroxyvitamin D concentrations predicted primary non-response and non-remission to infliximab and adalimumab in patients with Crohn’s disease.

## Methods

### Study Design

The Personalised Anti-TNF Therapy in Crohn’s Disease study (PANTS) is a UK-wide, multicentre, prospective observational cohort reporting the treatment failure rates of the anti-TNF drugs infliximab (originator, Remicade [Merck Sharp & Dohme, UK] and biosimilar, CT-P13 [Celltrion, South Korea]) and adalimumab (Humira [Abbvie, USA]) in anti-TNF-naïve patients with active luminal Crohn’s disease.^15^

Patients were recruited between February 2013 and June 2016 at the time of first anti-TNF exposure and studied for 12 months or until drug withdrawal. After 12 months, patients were invited to continue follow-up for a further 2 years. Eligible patients were aged ≥6 years with objective evidence of active luminal Crohn’s disease involving the colon and/or small intestine. Exclusion criteria included prior exposure to, or contraindications for the use of, anti-TNF therapy. The choice of anti-TNF was at the discretion of the treating physician and prescribed according to the licensed dosing schedule. Study visits were scheduled at the first dose, week 14, and at weeks 30 and 54. Additional visits were planned for infliximab-treated patients at each infusion and for both groups at treatment failure or exit.

For this analysis, we included all patients who had stored serum available from baseline visits and effectiveness outcomes. Patients were excluded from our effectiveness analysis if they had a stoma as Harvey Bradshaw Index (HBI) and short pediatric Crohn’s disease activity index (sPCDAI) scores have not been validated in these patient groups. Patients who were recruited into the study with normal prescreening visit 1 fecal calprotectin and C-reactive protein (CRP) levels, and where the only indication for anti-TNF was perianal disease, were also excluded.

### Outcomes

Treatment failure endpoints were primary non-response at week 14, non-remission at week 54, and adverse events leading to drug withdrawal.

We used composite endpoints using the HBI in adults and the sPCDAI in children, corticosteroid use, and CRP to define primary non-response (Figure S1). Remission was defined as CRP of ≤3 mg/L and HBI of ≤4 points (sPCDAI ≤15 in children), without corticosteroid therapy or exit for treatment failure.

Secondary outcomes included anti-TNF drug concentration measured at weeks 14 and 54 and the time to development of anti-TNF antibodies. Drug persistence was defined as the duration of time from initiation of anti-TNF therapy to exit from the study due to treatment failure.

Patients exited the study when they stopped anti-TNF therapy or had an intestinal resection regardless of surgical outcome. They were deemed to be in non-remission for subsequent time points. Patients who declined to participate in the 2-year extension or who exited the study for loss to follow-up, withdrawal of consent, or elective withdrawal of drug, including for pregnancy, were censored at the time of study exit and excluded from the denominator for subsequent analyses.

### Clinical Variables and Laboratory Analyses

Variables recorded at baseline by sites were demographics (age, sex, ethnicity, comorbidities, height and weight, and smoking status) and IBD phenotype and its treatments (age at diagnosis, disease duration, Montreal classification, prior medical and drug history, and previous Crohn’s disease-related surgeries). At every visit, disease activity score, weight, current therapy, and adverse events were recorded.

Blood and stool samples were processed through the central laboratory at the Royal Devon University Healthcare NHS Foundation Trust (https://www.exeterlaboratory.com/) for hemoglobin, white cell count, platelets, serum albumin, CRP, anti-TNF drug and antidrug antibody concentrations, and fecal calprotectin, respectively. Genotyping methods and the genetic analysis have been reported previously.^17^

#### 25-Hydoxyvitamin D concentrations

Serum 25-hydoxyvitamin D was measured in baseline samples between January 22, 2020, and March 20, 2020, using the Elecsys 25-hydoxyvitamin electrochemiluminescence immunoassay (Roche) using the Cobas 801 module on the Cobas 8000 analyzer.^22^ This competitive electrochemiluminescence assay uses a ruthenium-complexed vitamin D binding protein to capture vitamin D3 (25-OH) and vitamin D2 (25-OH). The local reference range defines vitamin D deficiency as <25 nmol/L, insufficiency as 25–50 nmol/L, and adequacy/sufficiency >50 nmol/L. Preanalytical stability of serum 25-hydroxyvitamin D following long-term sample storage at up to −40 °C has been demonstrated previously.^23,24^

#### TNF drug-level assays

The IDKmonitor free infliximab (K9655) and adalimumab (K9657) drug-level assays permit quantitative measurement of free therapeutic drug in serum.^15^ The assays follow a standard ELISA format using a specific monoclonal antidrug antibody fragment as a capture antibody and peroxidase-labeled anti-human IgG antibody as a detection antibody. The measuring range for both assays is 0.8–45 mg/L, with the absence of the drug being defined using a cutoff of <0.8 mg/L.

#### Drug-tolerant anti-TNF antibody assays

Total antidrug antibody concentrations were measured with IDKmonitor® ELISA assays (Immundiagnostik AG, Bensheim, Germany) performed on the Dynex DS2 ELISA robot (Dynex technologies, Worthing, UK). The Immundiagnostik (IDK) AG (Bensheim, Germany) IDKmonitor infliximab (K9654) and adalimumab (K9651) total antidrug antibody assays allow semi-quantitative measurement of both free and bound antidrug antibodies.^15^ A pretreatment acid dissociation step is used to separate antidrug antibodies from the therapeutic antibody. The assay then follows a standard ELISA format using a recombinant therapeutic antibody as a capture and detection antibody. The positivity thresholds for the infliximab and adalimumab assays are 9 and 6 AU/mL, respectively.^25^

### Study Size and Statistical Methods

The sample size calculation for the PANTS study has been reported previously.^15^ Here we included all patients who had sufficient stored serum from their baseline visit and had outcome data at week 14.

Statistical analyses were undertaken in R 4.1.3 (R Foundation for Statistical Computing, Vienna, Austria). All tests were 2-tailed and *P*-values of <0.05 were considered significant. We included patients with missing clinical variables in analyses for which they had data and have specified the denominator for each variable. Continuous data are reported as median and interquartile range (IQR), and discrete data as numbers and percentages. We performed univariable analyses using Fisher’s exact, Mann–Whitney *U*, and Spearman’s rank tests to identify differences in baseline characteristics between infliximab- and adalimumab-treated patients, and to determine categorical and continuous factors associated with vitamin D levels and the predefined clinical outcomes above. Multivariable logistic regression analyses were used to confirm factors independently associated with vitamin D deficiency. Rates of immunogenicity and drug persistence were estimated using the Kaplan–Meier method, and comparative analyses were performed using univariable and multivariable Cox proportional hazards regression. Further sensitivity analyses using fecal calprotectin at week 54 as an outcome and stratifying the cohort by vitamin D supplementation and/or corticosteroid treatments at baseline were undertaken.

## Results

### Participants

**Figure 1.**
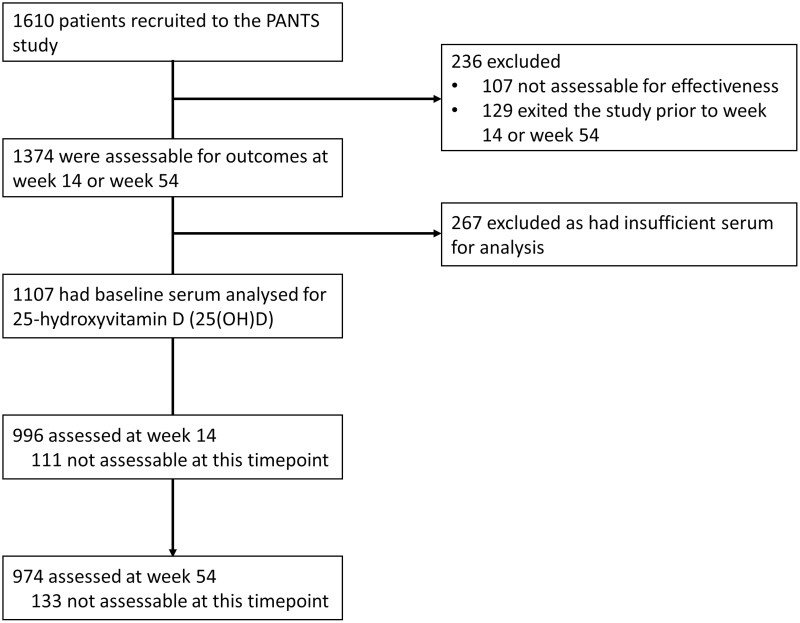
Study profile. Patients were not assessable when 1 or more of the key data items were missing.

Overall, 80.6% (1107/1374) of patients who participated in the PANTS study who were assessable for effectiveness were included: 659 (59.5%) were treated with infliximab (526 [47.5%] with originator infliximab, and 133 [12.0%] with biosimilar CT-P13) and 448 (40.5%) were treated with adalimumab (Figure 1). At baseline, 22.2% (246/1107) patients were receiving a form of vitamin D supplementation, of whom 52.8% (130/246) were prescribed corticosteroids. Differences between demographic and clinical characteristics of infliximab- and adalimumab-treated patients are shown in Table 1.

**Table 1.** Baseline demographic and clinical characteristics, stratified by anti-TNF.

Variable	Level	Infliximab	Adalimumab	Overall	*P*
*n*		659	448		
Sex	Female	50.68% (334/659)	53.57% (240/448)	51.85% (574/1107)	.358
	Male	49.32% (325/659)	46.43% (208/448)	48.15% (533/1107)	
Ethnicity	White	89.53% (590/659)	96.65% (433/448)	92.41% (1023/1107)	<.001
	South Asian	5.01% (33/659)	1.79% (8/448)	3.70% (41/1107)	
	Other	5.46% (36/659)	1.56% (7/448)	3.88% (43/1107)	
Anti-TNF	Adalimumab	0.00% (0/659)	100.00% (448/448)	40.47% (448/1107)	<.001
	CT-P13	20.18% (133/659)	0.00% (0/448)	12.01% (133/1107)	
	Remicade	79.82% (526/659)	0.00% (0/448)	47.52% (526/1107)	
Age at first dose of anti-TNF		30.03 (18.95–44.69)	38.60 (28.53–50.49)	33.26 (22.76–47.29)	<.001
Age at first dose of anti-TNF < 18		22.91% (151/659)	3.12% (14/448)	14.91% (165/1107)	<.001
Disease duration		2.08 (0.61–7.40)	2.80 (0.74–10.52)	2.26 (0.66–8.83)	.009
Montreal disease location	L1	28.09% (184/655)	33.26% (147/442)	30.17% (331/1097)	.306
	L2	24.12% (158/655)	21.72% (96/442)	23.15% (254/1097)	
	L3	46.72% (306/655)	44.34% (196/442)	45.76% (502/1097)	
	L4	1.07% (7/655)	0.68% (3/442)	0.91% (10/1097)	
Montreal L4 modifier		13.28% (87/655)	4.52% (20/442)	9.75% (107/1097)	<.001
Montreal disease behavior	B1	63.57% (417/656)	57.79% (256/443)	61.24% (673/1099)	<.001
	B2	25.91% (170/656)	36.79% (163/443)	30.30% (333/1099)	
	B3	10.52% (69/656)	5.42% (24/443)	8.46% (93/1099)	
Perianal disease		14.42% (95/659)	7.81% (35/448)	11.74% (130/1107)	<.001
Smoking history	Current	14.18% (92/649)	21.40% (95/444)	17.11% (187/1093)	<.001
	Ex	25.73% (167/649)	35.59% (158/444)	29.73% (325/1093)	
	Never	60.09% (390/649)	43.02% (191/444)	53.16% (581/1093)	
Body mass index (kg/m^2^)		22.49 (19.55–27.06)	24.28 (21.48–28.30)	23.29 (20.31–27.68)	<.001
Baseline immunomodulator use	TRUE	61.91% (408/659)	51.79% (232/448)	57.81% (640/1107)	.001
Baseline steroid use	TRUE	29.29% (193/659)	27.01% (121/448)	28.36% (314/1107)	.416
C-reactive protein (mg/L)		9.00 (3.00–23.00)	7.00 (2.00–14.00)	8.00 (3.00–19.00)	<.001
Fecal calprotectin (μg/g)		458.00 (186.50–898.25)	317.50 (141.50–629.00)	372.50 (163.50–761.50)	<.001
Hemoglobin (g/L)		125.00 (114.00–136.00)	131.00 (120.00–142.00)	127.00 (117.00–138.50)	<.001
Albumin (g/L)		39.00 (34.00–42.00)	40.00 (36.00–43.00)	39.00 (34.00–42.00)	.003
Harvey Bradshaw Index		6.00 (3.00–9.00)	5.00 (3.00–8.00)	5.00 (3.00–9.00)	.418
Short pediatric Crohn’s disease activity index		25.0 (15.0–50.0)	NA	25.0 (15.0–50.0)	NA

Similar to the whole cohort, there were significant differences at baseline between the infliximab- and adalimumab-treated patients, including in age, ethnicity, smoking, body mass index, disease duration, and disease behavior. Patients treated with infliximab had more active disease at baseline than patients treated with adalimumab, as evidenced by higher serum CRP and fecal calprotectin concentrations. At the initiation of anti-TNF treatment, immunomodulator use was higher in patients treated with infliximab compared to those treated with adalimumab, but there was no difference in the proportion of patients treated with corticosteroids.

### Baseline Factors Associated With Vitamin D Concentrations

**Figure 2.**
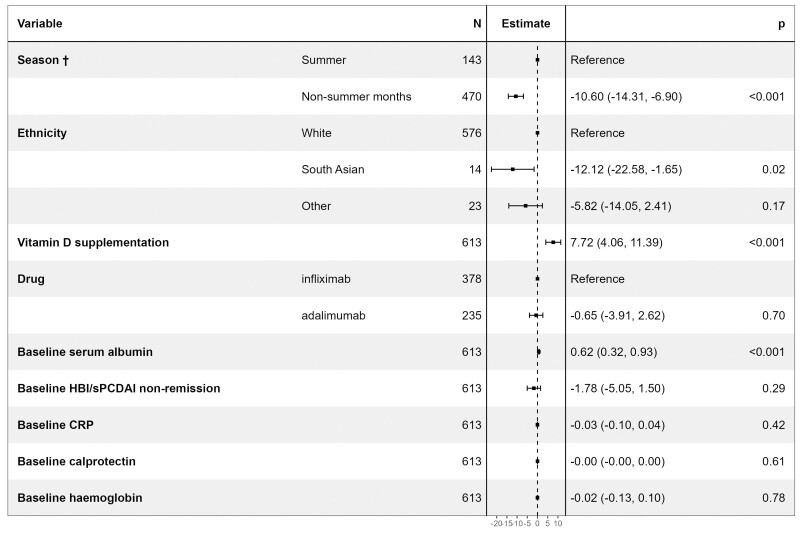
Forest plot showing the coefficients from a multivariable linear regression model of associations with pretreatment vitamin D concentrations. The resultant values represent the change in vitamin D concentrations associated with each variable. CRP, C-reactive protein; HBI, Harvey Bradshaw Index; sPCDAI, short pediatric Crohn’s disease activity index. ^†^Sampling during the summer was defined as a blood sample obtained in the months of June, July, and August.

Median [IQR] vitamin D concentrations were lower in patients subsequently treated with infliximab than adalimumab (39.0 nmol/L [29.0–56.0] versus 44.0 nmol/L [31.0–59.0], *P* = .02). The other univariable factors associated with vitamin D concentrations are shown in Table 2 and Table S2. Multivariable linear regression analysis confirmed that baseline sampling during non-summer months (Figure S2), South Asian ethnicity, lower serum albumin concentrations, and nontreatment with vitamin D supplementation were independently associated with lower vitamin D concentrations (Figure 2).

**Table 2.** Baseline demographic and clinical characteristics associated with vitamin D concentrations.

Categorical variables				
Variable	Level	*n*	Vitamin D (nmol/L)	*P*
Month of ­sampling	Non-summer	849	38.0 (27.0–54.0)	<.001
	Summer^a^	258	51.0 (39.0–65.0)	
Ethnicity	South Asian	41	30.0 (22.0–44.0)	.001
	White/Others	1066	42.0 (30.0–58.0)	
Pretreatment vitamin D supplementation	No	861	39.0 (28.0–55.0)	<.001
	Yes	246	50.0 (36.0–64.0)	
Drug	Infliximab	659	39.0 (29.0–56.0)	.021
	Adalimumab	448	44.0 (31.0–59.0)	

Continuous variables				
Variable			Spearman’s rho (*R*)	*P*
Serum albumin			0.18	<.001
Harvey Bradshaw Index			−0.07	.040
Short pediatric Crohn’s disease activity index			−0.33	.003
CRP^b^			−0.12	<.001
Fecal calprotectin^b^			−0.11	.003
Hemoglobin			0.10	.002

^a^Sampling during the summer was defined as a blood sample obtained for vitamin D analysis in the months of June, July, and August.

^b^Variables were log-transformed for analysis.

Abbreviation: CRP, C-reactive protein.

### Baseline Vitamin D Status and Clinical Outcomes

Overall, 17.0% (189/1107; 95% CI, 15.0–19.4) and 47.7% (528/1107; 95% CI, 44.8–50.6) patients had vitamin D deficiency and insufficiency, respectively. Primary non-response at week 14 and non-remission at week 54 occurred in 19.3% (116/600; 95% CI, 16.4–22.7) and 58.8% (351/597; 95% CI, 54.8–62.7) patients treated with infliximab and 25.3% (100/396; 95% CI, 21.2–29.8) and 65.3% (246/377; 95% CI, 60.3–69.9) of patients treated with adalimumab, respectively.

**Figure 3:**
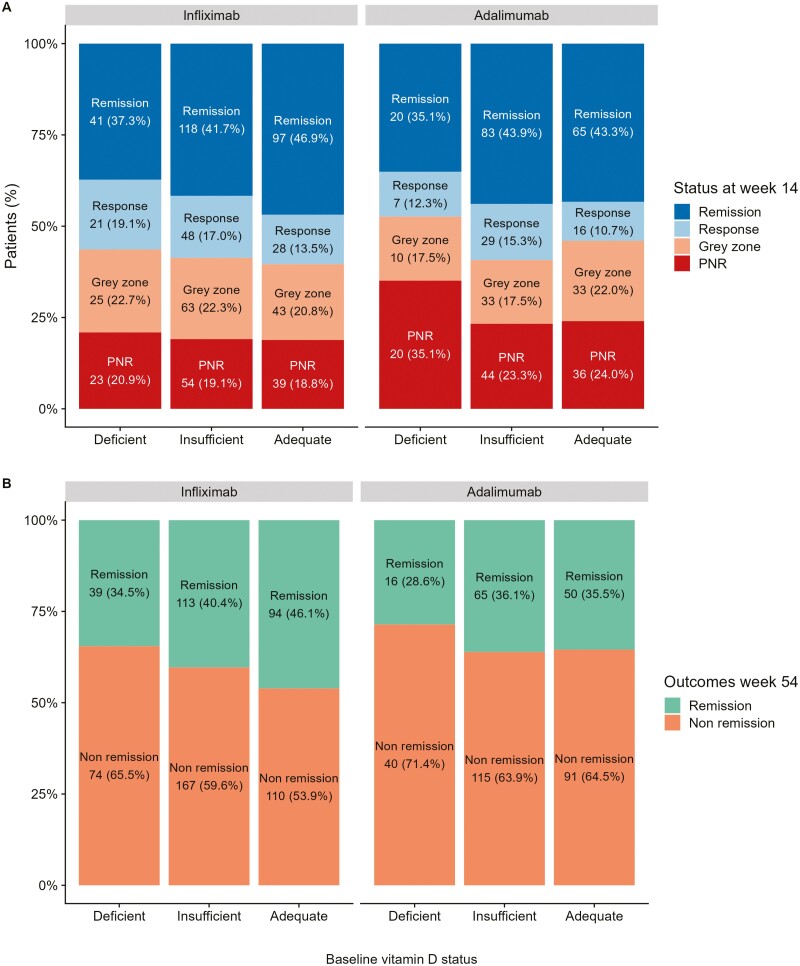
Proportion of patients stratified by their pretreatment vitamin D status and outcomes to anti-TNF at (A) week 14 and (B) week 54. Infliximab-treated patients on the left panel, and adalimumab-treated patients on the right panel. The number of patients experiencing each outcome is annotated in the plot, with the proportion in brackets (%). PNR, primary non-response.

Pretreatment vitamin D status did not predict response or remission status to anti-TNF therapy at week 14 (primary non-response: infliximab *P* = .89, adalimumab *P* = .18; remission: infliximab *P* = .19, adalimumab *P* = .38) or non-remission at week 54 (infliximab *P* = .13, adalimumab *P* = .58; Figure 3). Overall, there were no differences in median (IQR) vitamin D levels at baseline according to response or remission status at weeks 14 and 54, respectively (Figure S3).

In patients who continued in the study beyond week 14, there was no difference in drug persistence between patients with vitamin D deficiency (hazard ratio [HR] 0.98 [95% CI, 0.71–1.34], *P* = 0.89) or insufficiency (HR 1.08 [95% CI, 0.85–1.36], *P* = .54) at baseline compared to those with adequate concentrations.

### Sensitivity Analyses

In a subset of 47.1% (520/1107) of patients who had week 54 fecal calprotectin data, we found a weak negative correlation between vitamin D concentrations at baseline and fecal calprotectin concentrations at week 54 (Rho = −0.09, *P* = .04).

Of the 28.4% (314/1107) patients treated with corticosteroids at baseline, 41.4% (130/314) were receiving concurrent vitamin D supplementation. Vitamin D concentrations were higher in those receiving vitamin D supplementation compared to those who were not (50.0 nmol/L [36.3–64.8] vs 36.0 [25.8–48.0], *P* < .001); however, there was no difference in primary non-response rates at week 14 (35.3% vs 32.7%, *P* = .70) or non-remission at week 54 (65.5% vs 63.1%, *P* = .71).

We then excluded from the whole cohort, all patients who were receiving vitamin D supplementation at baseline. Of 773 patients remaining, pretreatment vitamin D status was not associated with primary non-response at week 14 (*P* = .15) or non-remission at week 54 (*P* = .26).

### Anti-TNF Drug Concentrations and Time to Immunogenicity

We observed a weak positive correlation between pretreatment vitamin D concentration and anti-TNF drug concentrations at week 14 (infliximab: Rho = 0.10, *P* = .03; adalimumab: Rho = 0.20, *P* < .001); however, when we included the factors previously associated with week 14 drug level, vitamin D concentrations were not independently associated in our multivariable models (Figure S4). We did not demonstrate associations with infliximab or adalimumab drug concentrations at week 54.

**Figure 4.**
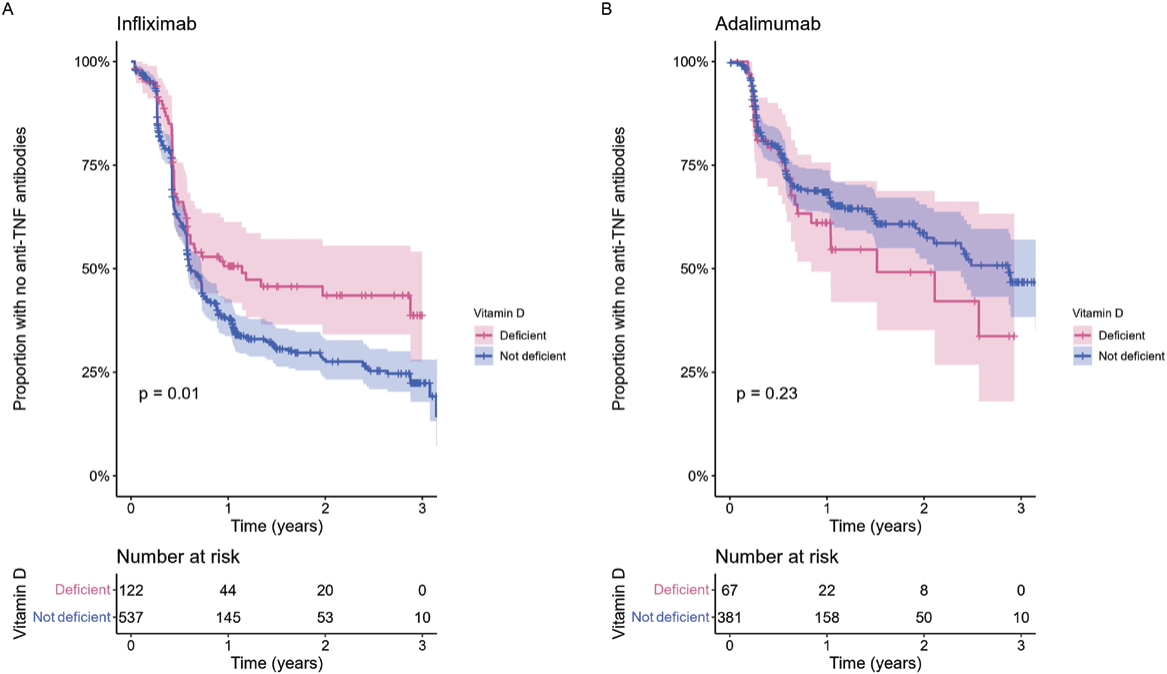
Kaplan–Meier estimates of time to the development of anti-TNF antibodies in patients stratified by pretreatment vitamin D status. Infliximab-treated patients are shown in (A) and adalimumab-treated patients in (B). *P*-values calculated using the log-rank test. Shaded regions represent the 95% CI.

**Figure 5.**
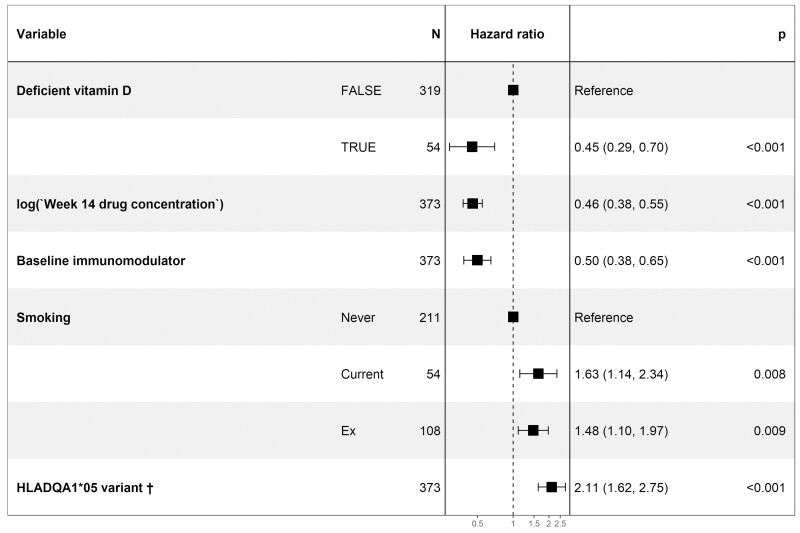
Forest plot showing the hazard ratio of the factors associated with time to the development of anti-TNF antibodies in infliximab-treated patients. ^†^Patients with either 1 or 2 copies of the allele were considered to have carriage of the HLA-DQA1*05 variant.

The estimated proportion of patients who developed antidrug antibodies for the first, second, and third years was 64.4% (95% CI, 60.0–68.4), 69.6% (95% CI, 64.9–73.6), and 78.4% (95% CI, 69.1–84.9) in infliximab-treated patients; and 36.9% (95% CI, 31.5–41.8), 45.8% (95% CI, 38.7–52.1), and 55.1% (95% CI, 45.6–62.9) in adalimumab-treated patients, respectively. Time to immunogenicity was longer in patients with vitamin D deficiency in infliximab-treated (HR 0.69 [95% CI, 0.52–0.91], *P* = .01]), but not adalimumab-treated (HR 1.29 [95% CI, 0.85–1.95], *P* = .23) patients (Figure 4). Multivariable analysis, including drug concentration at week 14, immunomodulator use, smoking, and carriage of the HLA-D1A1*05 variant that we have previously reported to be associated with time to immunogenicity in this cohort, confirmed that vitamin D deficiency was independently associated with a longer time to immunogenicity in infliximab-treated patients (Figure 5).

## Discussion

### Key Results

Vitamin D deficiency is common in patients with active Crohn’s disease. Unlike previous studies, pretreatment serum 25-hydroxyvitamin D concentrations did not predict primary non-response, non-remission, or anti-TNF drug persistence. Vitamin D deficiency was, however, associated with a longer time to immunogenicity in patients treated with infliximab.

### Interpretation

Our observation that 17% and 48% of patients had vitamin deficiency and insufficiency, respectively, is consistent with previous estimates in patients with active IBD and almost double that compared of healthy controls.^3–5^ Moreover, our model of 25-hydroxyvitamin D concentration confirmed independent associations with baseline sampling during non-summer months, South Asian ethnicity, lower serum albumin concentrations, and nontreatment with vitamin D supplementation further validates our findings against clinical outcome. Unlike Winter et al.,^19^ Zator et al.,^20^ and Xia et al.,^21^ in their small mixed cohorts, we did not see associations with primary non-response, durability of IBD therapy or remission at week 54, respectively. The major criticisms of these studies are measurement bias (to be included patients needed to have had vitamin D measured); the retrospective assessment of remission; how disease severity was controlled for; and the lack of data relating to concomitant corticosteroid therapy or vitamin D supplementation.^26^ This is the first prospective study with a large enough sample size to adjust for potential confounders to examine the association between pretreatment vitamin D status/concentration and clinical outcomes in patients with Crohn’s disease treated with anti-TNF therapy. Our negative findings are consistent with the findings from a small (56 Crohn’s disease and 12 ulcerative colitis) prospective study reported by Santos-Antunes et al.^27^ In our sensitivity analyses, we did not observe any association between pretreatment vitamin D concentrations and clinical outcomes when we stratified our data by vitamin D supplementation in patients who were treated with corticosteroids at baseline. Our weak association with fecal calprotectin suggests that vitamin D supplementation might have, at best, a modest immunoregulatory role in anti-TNF therapy. Overall, however, and unlike previous reports, our data provide no additional justification for the use of vitamin D supplementation in anti-TNF treatment over current indications.

The finding that vitamin D deficiency was lower in infliximab-treated patients is likely to be explained by more active disease at baseline evidenced by a higher serum CRP and fecal calprotectin observed in infliximab- compared to adalimumab-treated patients in this real-world study.

Our observation that the time to immunogenicity was longer in patients with vitamin D deficiency is of interest. Vitamin D has a central role in antigen presentation and T cell function, with effects on immune tolerance in adaptive immune responses.^28^ While vitamin D deficiency did not predict primary non-response to anti-TNF treatment, whether low vitamin D levels protect against the development of antidrug antibodies requires further study.

### Limitations and Generalizability

We acknowledge the following limitations. We accept that our data would have been strengthened by endoscopic outcomes or cross-sectional imaging. However, in PANTS,^15^ we observed a significant association between clinical outcomes at weeks 14 and 54 with fecal calprotectin, which correlates closely with endoscopic findings. In our sensitivity analysis, we did not observe a clinically useful correlation (Rho = −0.09, *P* = .04) between pretreatment vitamin D concentrations and calprotectin. The addition of cross-sectional imaging would have strengthened our data in those with a disease affecting the small bowel, but less so in those with disease affecting the large bowel only. Because our stored samples are slowly being exhausted, in particular in children,^16^ we accept there was some missingness in our cohort. We may have been underpowered to detect associations between vitamin D concentrations and time to the development of anti-adalimumab antibodies, because immunogenicity events were less common and fewer in adalimumab- than in infliximab-treated patients who were vitamin D deficient at baseline.

Our findings are likely to be generalizable to other patients with Crohn’s disease, at least at latitudes similar to those in the United Kingdom. It is possible that vitamin D deficiency in patients with IBD at different latitudes will be less prevalent and whether our findings are generalizable in these populations remain unknown. It is perhaps less likely that vitamin D deficiency influences anti-TNF treatment responses in rheumatoid arthritis, ankylosing spondylitis, psoriatic arthritis, psoriasis, hidradenitis suppurativa, and uveitis because these conditions are not associated with intestinal malabsorption of dietary vitamin D. Whether our findings are generalizable to other anti-TNF drugs, including certolizumab, golimumab, etanercept, and other biologicals, is unknown.

## Conclusions

Vitamin D deficiency is common in patients with active Crohn’s disease. Unlike previous studies, pretreatment serum 25-hydroxyvitamin D concentration did not predict primary non-response to anti-TNF treatment at week 14 or non-remission at week 54.

## Supplementary Material

otad026_suppl_Supplementary_Material

## Data Availability

Individual participant deidentified data that underlie the results reported in this article will be available immediately after publication for a period of 5 years. The data will be made available to investigators whose proposed use of the data has been approved by an independent review committee. Analyses will be restricted to the aims in the approved proposal. Proposals should be directed to tariq.ahmad1@nhs.net. To gain access, data requestors will need to sign a data access agreement.
